# Correlations between EEG and intestinal electrical stimulation

**DOI:** 10.1515/tnsci-2022-0256

**Published:** 2022-12-06

**Authors:** Nora Vanessa de Camp, Jürgen Bergeler

**Affiliations:** Department of Behavioral Physiology, Institute for Biology, Humboldt-Universität zu Berlin, Berlin, Germany; Medical Center of the Johannes-Gutenberg University Mainz, Visceral Surgery Unit, Mainz, Germany

**Keywords:** electroencephalography, intestinal electrical stimulation, phase amplitude coupling, pig, brain-gut-axis

## Abstract

Many diseases affect the autonomous nervous system and the central nervous system simultaneously, for example Parkinson’s disease or irritable bowel syndrome. To study neurophysiologic interactions between the intestinal electrical activity and the electroencephalography (EEG) pattern of the brain, we combined intestinal electrical stimulation (IES) and non-invasive telemetric full-band DC EEG recordings in an acute pig-model. Intestinal motility was monitored with accelerometers. Brain activity was analyzed with regard to network driven phenomena like phase amplitude coupling (PAC) within two time-windows: 1 min after IES (early response) and 3 min after stimulation (late response). Here we present the results for two stimulation sites (small intestine, colon) and two parietal scalp-EEG channels (right and left somatosensory cortex region). Electrical stimulation consisted of a 30 or 130 Hz pulse. In summary, the PAC modulation index at a parietal EEG recording position is decreased after IES. This effect is in line with an inhibitory effect of our IES protocol regarding peristalsis. The surprisingly strong effects of IES on network driven EEG patterns may be translated into new therapeutic techniques and/or diagnostic tools in the future. Furthermore, analytic tools, operating on sparse datasets, may be ideally suited for the integration in implantable intestinal pacemakers as feedback system.

## 1 Introduction

Intestinal electrical stimulation (IES) is an emerging therapeutic option for intestinal diseases with impairment of peristalsis [1]. The same is true for electrophysiological stimulation techniques, especially deep brain stimulation in the case of central nervous diseases, for example, depressive disorders and Parkinson’s disease [2,3].

The above two examples are also representative disorders which are by far not restricted to the central nervous system (CNS) [4,5]. For Parkinson’s disease, it is hypothesized whether this pathology may have its origin in the intestinal system rather than the brain [6]. In order to understand such pathologic pathways, it may be important to understand the bilateral connection of the intestinal system and the brain [5,7]. The enteric nervous system (ENS) is part of the autonomic nervous system [8] with associated pacemakers in the plexus [9] and a closed circuit which is not dependent on inputs from the CNS. The other two branches of the autonomic nervous system are the sympathetic nervous system and the parasympathetic nervous system which have their origin in the brainstem region and the spinal cord to control vital functions, for example, breathing, and they can both modulate the ENS. Neurophysiologic signal transfer between CNS and ENS can run along these axes, sympathetic and in the case of the parasympathetic axis mostly the vagal nerve [5]. There are several examples for important inputs and control mechanisms from the brain to the intestinal system, for example, deliberate control of defecation, the start of peristalsis, and salivation in the case of visual and other sensory stimuli in the context of food perception [10]. The other way is also realized, as for example, the induction of food seeking behavior by signals from the intestinal system as well as other physiologic parameters [11]. The main question of the following work is whether IES in the intestinal tract has any kind of influence on the non-invasive electroencephalography (EEG) pattern, especially on network driven phenomena. The second question is whether these phenomena can be detected with few trials, in contrast to evoked potentials, where many repetitions under laboratory conditions are necessary to extract these tiny electrical fluctuations of the cortex.

Phase amplitude coupling (PAC) is one such network-driven phenomenon, which is not restricted to the brain but instead can also be observed in the pacemaker network to generate peristaltic movements of the intestinal tract [12]. PAC is a result of the interplay between different oscillations which drive an amplitude modulation. A “wave” or “oscillation” represents the synchronized activity of an ensemble of neurons or electrically active cells, quasi the envelope of neuronal group activity. The phenomenon occurs, if a slower wave is in a certain phase relation to a faster wave. This synchrony can lead to an amplitude modulation, typically of the faster wave [13]. The relational nature of this phenomenon makes it relatively robust against noise or artifacts [14]. PAC has been used to study vigilance states [15] or to identify the epileptogenic focus in human patients with temporal lobe epilepsy [16]. But as mentioned beforehand, the phenomenon also plays a role in peristaltic rhythm generation in the intestinal tract with its plexus [12].

From former studies, it is known that IES can influence the electrophysiology of the brain even in cortical regions. Frieling et al. [17] recorded evoked potentials in response to electrical stimulation of the rectosigmoid colon in human volunteers (similar studies: [18]) and in a similar experimental setup, spinal evoked potentials have been recorded additionally [19]. Other studies with human volunteers focused on the central location of pain generation after IES of the sigmoid colon [20,21] and the modulation of pain after administration of morphine [22]. Studies in cats revealed sleep related cortical potentials which are correlated with duodenal activity [23] and suggest synchronizing central effects of intestinal activity via splanchnic nerves [24]. In some diseases, as for example irritable bowel syndrome (IBS), the pain response after colonic ES is changed in different brain regions [25]. A relationship between brain activity and IES has also been investigated in human patients regarding constipation and rectal hyposensitivity [26], hypersensitivity [27], and changes in central pain perception in pancreatitis [28]. Most of the studies used evoked potentials to find central effects of IES. Evoked potentials require usually lots of repetition trials for analysis. Here we focus on PAC to study central effects of IES with only few trial repetitions, which may be closer to the clinical context. Another difference to most of the studies is that we used an acute pig model and the IES occurred at the serosal surface facing to the abdominal cavity instead of the inner intestinal wall, facing the intestinal lumen. The serosal surface is the location, where intestinal pacemakers can be implanted. The stimulation pattern of these intestinal pacemakers is still more or less empiric even though enormous technical possibilities already exist [29]. Robust analytic tools which work on sparse datasets may lower the threshold to study central effects of IES in different disease states in the clinical context. First, this may be an important factor to understand disease onset as well as general patho-electrophysiology. Second, it may be an interesting future diagnostic as well as therapeutic tool, also in combination with intestinal implants.

## 2 Materials and methods

### 2.1 Animal experiments

Three pietrain pigs (30 ± 5 kg body weight, male, 10 weeks old) were premedicated with azaperone intramuscularly (2 mg/kg weight; Stresnil, Janssen-Cilag), midazolam (0.3–0.54 mg/kg weight; Ratiopharm), and atropine (0.033 mg/kg weight) before initiation of anesthesia with intravenous sodium thiopental (5 mg/kg bolus followed by an intravenous infusion (10 mg/kg/h). The pigs were mechanically ventilated with a Dräger respirator Servo 900B (oxygen-air: FiO_2_ 0.27; pCO_2_ controlled) after intubation. Prior to intubation, Piritramid was administered intravenously (7.5 mg bolus) and maintained by an intravenous infusion (0.25 mg/kg/h). Arterial and central venous lines were introduced via the femoral artery and vein. A suprapubic catheter was introduced into the bladder. Heart rate and oxygen saturation were continuously measured using electrocardiogram, pulse oximetry, and capnometry. Ventilation was adjusted according to repeated blood gas analysis. For volume substitution, Ringer solution (10 mL/kg/h) was constantly infused during the operation. The experimental procedure was kept constant, throughout the experiments. Stimulation parameters are as follows: amplitude: 30 mA, stimulation time 30 s, pause between two stimulation runs: 3 min, pulse width 1,000 or 500 µs, and frequency 30 or 130 Hz (the same setup as in ref. [30]). Each stimulation run consisted of 4 stimulations, respectively. One stimulation run per gastrointestinal location and per pig, which means that for example four stimulations, interspersed with 3 min pause, were done at the small intestine, and then after a longer pause, again 4 stimulations were done at the colon. The stimulation device (experimental setup of the company Inomed GmbH) was used in combination with hook wire electrodes (Inomed GmbH). Stimulation was applied to the small intestine (different positions have been pooled: duodenum, jejunum, ileum) and to the colon (colon ascendens and colon descendens pooled). For electrical stimulation, hook-wire electrodes were fixed to the outer serosal layer of the intestinal wall, near to the longitudinal muscle layer. After running of each stimulation, a 3 min pause was included. Each change in the stimulation electrode was preceded by a new control recording, in order to compensate for possible general physiologic changes during the relatively long period of experimentation. The stimulation (Table 1) was applied with this strict timing protocol, regardless of the quality of the EEG.

**Table 1:** Stimulation protocol

30 s stim	3 min pause	New IES location, 5 min pause	30 s stim	3 min pause
EEG and acceleration recording		EEG and acc control recording	EEG and acceleration recording	

The peristalsis was continuously monitored with 7 accelerometers, which were glued (vetbond, 3M Corporation, Saint Paul, Minnesota, USA) to the outer intestinal wall at different locations (liver, gut, 2xduodenum, 2xsmall intestine (duodenum = proximal, ileum = distal), colon (ascendens = proximal, *n* = 7) (for more details refer de Camp et al., 2018). The accelerometers have not been placed in direct vicinity to the stimulation electrode. The sensor MXR9500G/M (Memsic Inc., Andover, Massachusetts, USA) is DC coupled and can detect very slow movements. The frequency range is from 0 to 17 Hz with a standard −3 dB amplitude decrease. Common accelerometers start with 0.1 up to 1 Hz, which may be too high to detect slow peristaltic movements. The operation of this Memsic accelerometer is based on the symmetry of an internal thermal field. The liver represents a possible impact of artificial ventilation due to its sternal position.

### 2.2 EEG recordings

EEG subdermal needle electrodes (NeuroDart, distributed by GVB-geliMed KG, Germany) were placed at a parietal position on each hemisphere (one electrode on each hemisphere, which means two recording electrodes). The electrode position below the ears, laterally on the forehead, represents a part (snout representation and secondary somatosensory cortex) of the area of the somatosensory cortex [31]. The ground electrode was placed between the ears and the reference on the nose of the pig. All wires were connected to a telemetric full band DC EEG amplification device (made by Jürgen Bergeler and Nora de Camp, Berlin, Germany). The telemetry system was based on an nRF51822 (Nordic Semiconductors) integrated circuit (IC). This IC contains the CPU and the transceiver to transmit data wirelessly in the ISM-band (Industrial-Scientific-Medical) with 2.4 GHz frequency range. The analog signal was measured and preprocessed by the IC ADS1298 manufactured by Texas Instruments. This IC furthermore manages the analog-digital conversion, controlled by the CPU, whereby we adjusted the frequency of the sampling rate to 1,000 samples per second.

The raw signal was received, re-transformed in an analog signal, and then combined with other analog channels, for example, the stimulus artifact of the IES. This effort was made to avoid timing errors between the different analog channels. Finally, all analog signals were synchronously digitized with 1,000 samples per second by a picolog AD converter system (Pico Technology Limited, UK).

### 2.3 Statistics and data analysis

For data analysis MATLAB (2016b, The MathWorks Inc.) was used as well as the open source software Brainstorm (Version 3.200519, [32]). Only EEG data without artifacts were taken into account, which means, some trials have been rejected. The running EEG had no influence on the stimulation protocol. We used the function “PAC,” implemented in Brainstorm with the method mean vector length, proposed by Canolty and Knight [33]. This method is based on network phenomena where different EEG bands are synchronized with each other and the synchronization is accompanied by amplitude modulation of the faster frequency (https://neuroimage.usc.edu/brainstorm/Tutorials/TutPac). We analyzed the first and third minute after IES, called early response and late response. As control, we used trials without stimulation. Each stimulation run was interrupted by a pause of 3 min. Each change in the stimulation electrode was preceded by a new control recording.

For each time window after stimulation and for each hemisphere and animal, we calculated the maximal PAC value (max PAC) with the PAC function implemented in the program Brainstorm (https://neuroimage.usc.edu/brainstorm/Tutorials/TutPac). Band Nesting was set to 2–12/s and Band Nested was set to 13–150/s. Total number of frequency bins was set to zero and number of signals to process at once to one. These values were also collected for a control group without stimulation. The three groups were compared with a Kruskal–Wallis test (MATLAB function “kruskalwallis”). The stats matrix of the Kruskal–Wallis test can be used directly for the multiple comparison test, which was used to calculate exact statistical relations between groups (MATLAB function “multcompare,” alpha = 0.1), including the multiple testing problem-correction. The results of this test give a confidence interval (CI) as well as a *p*-value. If the CI does not contain zero, the difference may be statistically significant, depending on the *p*-value.

The phase locking value (Measure: Magnitude) and the amplitude envelope correlation were calculated with brainstorm. Frequency bands for the Hilbert transform were chosen as:

delta/2, 4/mean value

theta/5, 7/mean value

alpha/8, 12/mean value

beta/15, 29/mean value

gamma1/30, 59/mean value

gamma2/60, 90/mean value

gamma3/91, 130/mean value

gamma4/131, 180/mean value

All correlations were calculated with the Matlab function “corr,” type “Spearman.” To calculate the correlation between two EEG channels, we calculated each EEG band separately. EEG raw data were filtered with digital butterworth filters with a custom written Matlab script. The filter was designed with the function butter (*n* = 3rd order). We calculated the normalized cutoff frequency (Wn) for EEG bands delta [0–4 Hz], theta [4–8 Hz], alpha [8–13 Hz], beta [13–30 Hz], low gamma [30–80 Hz], and high gamma [80–120 Hz]. Wn is a number between 0 and 1, where 1 corresponds to the Nyquist frequency which is half the sampling rate (here: 500 Hz).

The numerator and denominator values (IIR filter), achieved with the function butter, were used with the Matlab function filtfilt to filter the EEG data. For the delta EEG band (0–4 Hz), a lowpass was used. We extracted all other EEG frequency bands with a bandpass filter design.

Furthermore, we calculated the correlation between PAC value and corresponding time range acceleration of the colon and small intestine with the Matlab function “corr,” Type “Spearman.”

The intestinal motility was calculated as norm of the *x*-, *y*-, and *z*-acceleration values in *g*: (*x* ^2^ + *y* ^2^ + *z* ^2^)^(1/2)^. Then, we calculated the mean norm acceleration for each time period, which was used for the EEG analysis.


**Ethical approval:** The research related to animals’ use has been complied with all the relevant national regulations and institutional policies for the care and use of animals. All procedures were approved by the local ethics committee (#23 177-07/G 17-1-008), and followed the European and the German national regulations (European Communities Council Directive, 86/609/ECC; Tierschutzgesetz). All animal procedures were performed in accordance with the (Medical Center of the Johannes Gutenberg-University Mainz) animal care committee’s regulations.

## 3 Results

**Figure 1:**
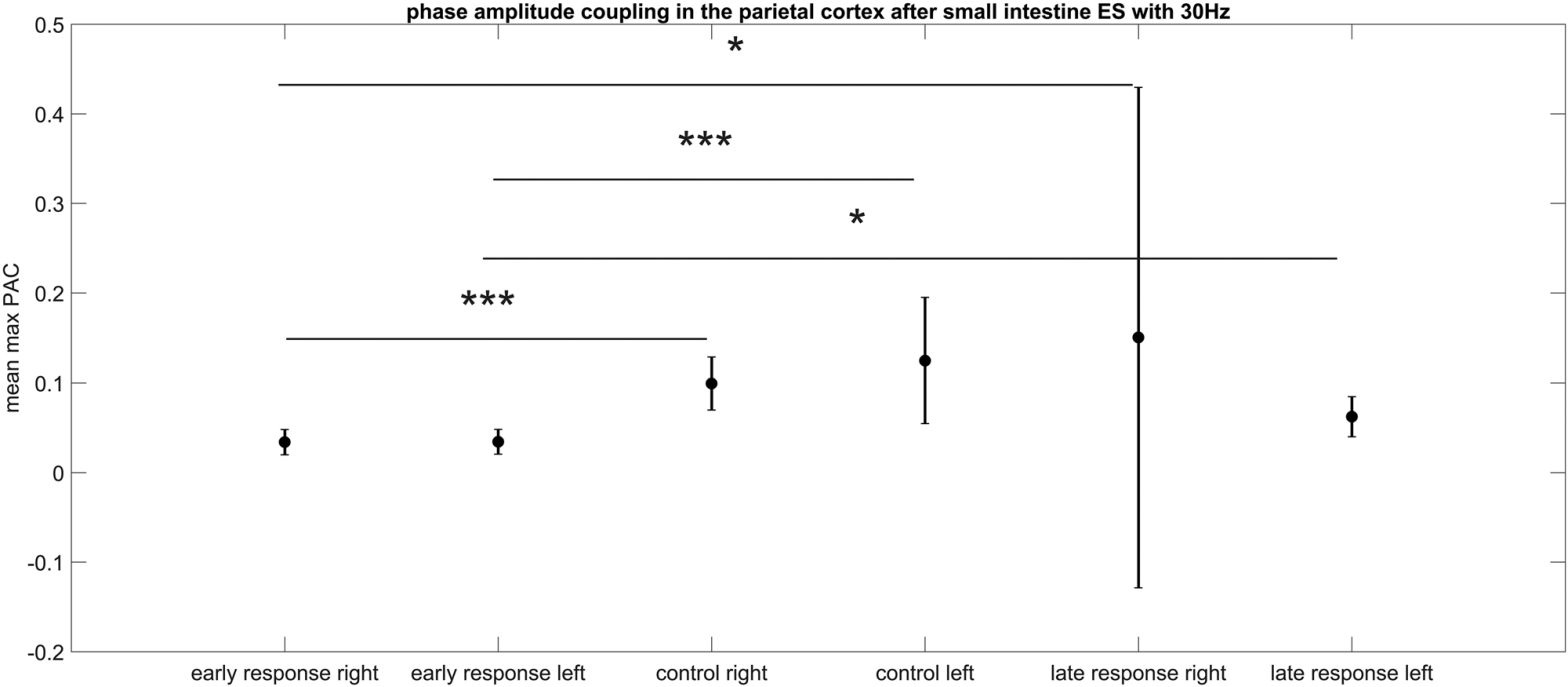
PAC after small intestine electrical stimulation with 30 Hz. The error bar represents standard deviation. The early response max PAC of the left as well as the right brain hemisphere (parietal recording position) are statistically significantly lower in comparison to the corresponding control and the corresponding (from the same hemisphere) late responses. This indicates a lower degree of PAC within the somatosensory cortex region directly after the electrical stimulation of the small intestine with a 30 Hz pulse to the outer serosal layer.

After electrical stimulation of the small intestine with a pulse of 30 Hz frequency, we found a statistically significant difference between the EEG maximal PAC value (max PAC, *n* = 5 stimulations) of the first minute after stimulation and the control condition (without IES, *n* = 5 control recordings without stimulation, Figure 1). The mean max PAC value is decreased during the time range directly after electrical stimulation (early response) in comparison to the control (right: CI −26.91 to −9.99] and *p* = 2.3 × 10^−5^, left: CI −28.42 to −11.49] and *p* = 3.9 × 10^−6^). There is also a tendency that the max PAC 3 min after stimulation (late response) on the left parietal electrode position (*p* = 0.03) as well as the right parietal electrode position (*p* = 0.02) is different from the early response (Tables 2 and 3, statistically significant differences are shaded light grey, differences which are still significantly different but with weaker *p*-value are shaded in blue). In the case of the late response, the max PAC value is increased in comparison to the early response (Figure 1).

**Table 2:** PAC left parietal electrode position

Group	Group	Lower CI	Estimate	Upper CI	*p*-value
1	2	−28.42	−19–95	−11.49	<0.001
1	3	−18.92	−10.45	−1.99	0.03
2	3	1.04	9.50	17.96	0.06

1 = first time window/fast response, 2 = without stimulation, 3 = late response, IES small intestine (30 Hz frequency).

**Table 3:** PAC right parietal electrode position

Group	Group	Lower CI	Estimate	Upper CI	*p*-value
1	2	−26.91	−18.45	−9.99	<0.001
1	3	−19.60	−11.14	−2.68	0.02
2	3	−1.14	7.32	15.78	0.18

1 = first time window/fast response, 2 = without stimulation, 3 = late response, IES small intestine (30 Hz frequency).

**Figure 2:**
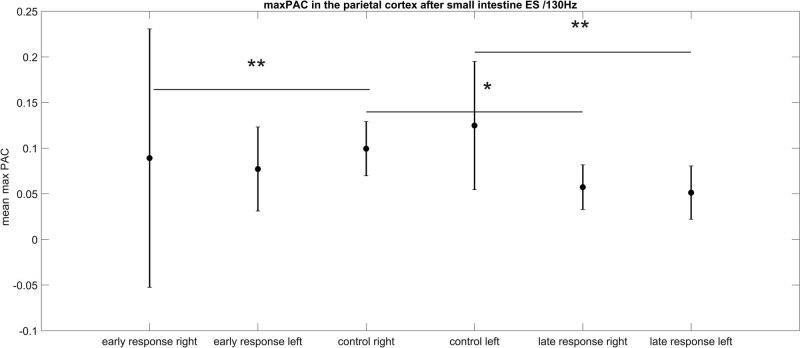
PAC after small intestine electrical stimulation with 130 Hz. The error bar represents standard deviation. A statistically significant reduction in the max PAC value can be detected for the late response of both hemispheres in comparison to the corresponding control. In contrast, the max PAC value of the early response is elevated only for the right hemisphere in comparison to the corresponding control.

We did the same analysis for a small intestine ES stimulus of 130 Hz frequency. The results of the statistical test are shown in Tables 4 and 5. For both hemispheres (left and right parietal electrodes), the max PAC values are statistically significantly different between the control group without stimulation and the late response, 3 s after IES on the small intestine (right: CI 3.76–23.44 and *p* = 0.013, left: CI 8.99–28.67 and *p* = 0.00025). For the right hemisphere, there is additionally a statistically significant difference between the early response, directly after IES at the small intestine and the control group without IES (CI –24.54 to –4.86 and *p* = 0.0062) (Figure 2). The max PAC values after IES again decreased in comparison to the control situation.

**Table 4:** PAC left parietal electrode position

Group	Group	Lower CI	Estimate	Upper CI	*p*-value
1	2	−19.91	−10.07	−0.23	0.09
1	3	−1.07	8.77	18.61	0.16
2	3	8.99	18.83	28.67	0.00025

1 = first time window/fast response, 2 = without stimulation, 3 = late response, IES small intestine (130 Hz frequency).

**Table 5:** PAC right parietal electrode position

Group	Group	Lower CI	Estimate	Upper CI	*p*-value
1	2	−24.54	−14.70	−4.86	0.0062
1	3	−10.94	−1.10	8.74	0.97
2	3	3.76	13.60	23.44	0.013

1 = first time window/fast response, 2 = without stimulation, 3 = late response, IES small intestine (130 Hz frequency).

**Figure 3:**
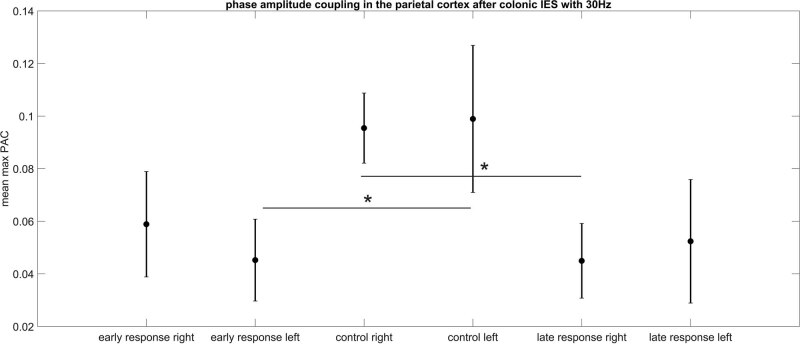
PAC after colon electrical stimulation with 30 Hz. The error bar represents standard deviation. The max PAC value is elevated for the early response and left hemisphere and decreased for the right hemispheric late response.

Tables 6 and 7 show the results of the Kruskal–Wallis test for a colon stimulation with a 30 Hz frequency IES pulse (Figure 3). On the left hemisphere (parietal electrode position), only the difference between the early response and the control is statistically significant different (CI –13.4 to –1.8 and *p* = 0.02). The early response max PAC value decreased in comparison to the control situation. For the right hemisphere, only the late response is statistically significantly different from the control (CI 2.4–14] and *p* = 0.01). In this case, the max PAC value decreased in comparison to the control EEG measurement without IES.

**Table 6:** PAC left parietal electrode position

Group	Group	Lower CI	Estimate	Upper CI	*p*-value
1	2	−13.40	−7.60	−1.80	0.02
1	3	−7.20	−1.40	4.40	0.87
2	3	0.40	6.20	12.00	0.07

1 = first time window/fast response, 2 = without stimulation, 3 = late response, IES colon (30 Hz frequency).

**Table 7:** PAC right parietal electrode position

Group	Group	Lower CI	Estimate	Upper CI	*p*-value
1	2	−11.40	−5.60	0.20	0.12
1	3	−3.20	2.60	8.40	0.63
2	3	2.40	8.20	14.00	0.01

1 = first time window/fast response, 2 = without stimulation, 3 = late response, IES colon (30 Hz frequency).

**Figure 4:**
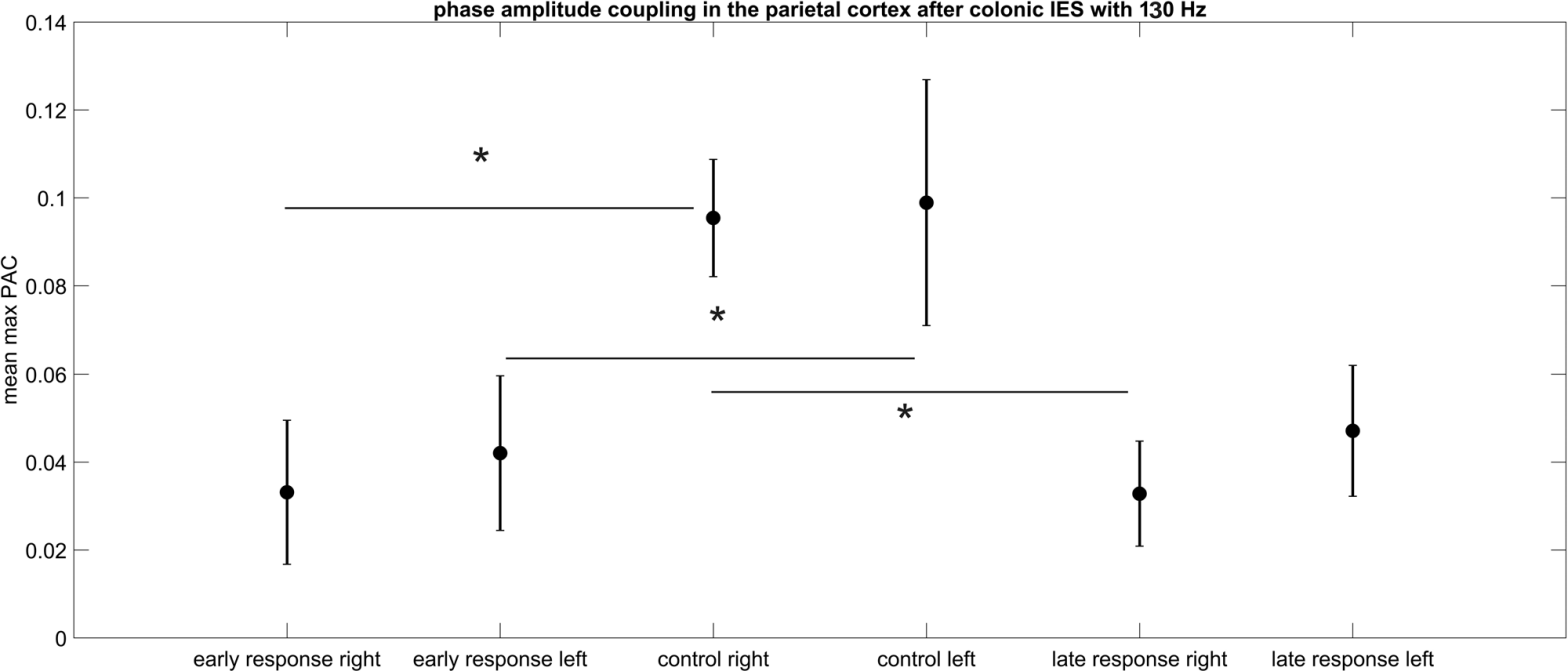
PAC after colon electrical stimulation with 130 Hz. The error bar represents standard deviation. The early response max PAC values of both hemispheres are statistically significantly decreased in comparison to the corresponding controls, respectively. In tendency, the late responses are also decreased in comparison to the control but only the decrease for the right hemisphere late response max PAC value is statistically significant.

Finally, in Tables 8 and 9, we show the results of the Kruskal–Wallis test and following multiple comparison test for a colon stimulation with a 130 Hz frequency stimulation pulse. Again, the max PAC value decreased after stimulation in comparison to the control (Figure 4). For the left hemisphere, a statistically significant difference can only be seen between control (without IES) and the early response after electrical colon stimulation (CI –13.2 to –1.6 and *p* = 0.02). For the right hemisphere, both, the early (CI –13.6 to –2 and p = 0.02) and the late response (CI [1.4–13] and *p* = 0.03) are statistically significantly different in comparison to the control.

**Table 8:** PAC left parietal electrode position

Group	Group	Lower CI	Estimate	Upper CI	*p*-value
1	2	−13.20	−7.40	−1.60	0.02
1	3	−6.80	−1	4.80	0.93
2	3	0.60	6.40	12.20	0.06

1 = first time window/fast response, 2 = without stimulation, 3 = late response, IES colon (130 Hz frequency).

**Table 9:** PAC right parietal electrode position

Group	Group	Lower CI	Estimate	Upper CI	*p*-value
1	2	−13.60	−7.80	−2.00	0.02
1	3	−6.40	−0.60	5.20	1.00
2	3	1.40	7.20	13.00	0.03

1 = first time window/fast response, 2 = without stimulation, 3 = late response, IES colon (130 Hz frequency).

**Figure 5:**
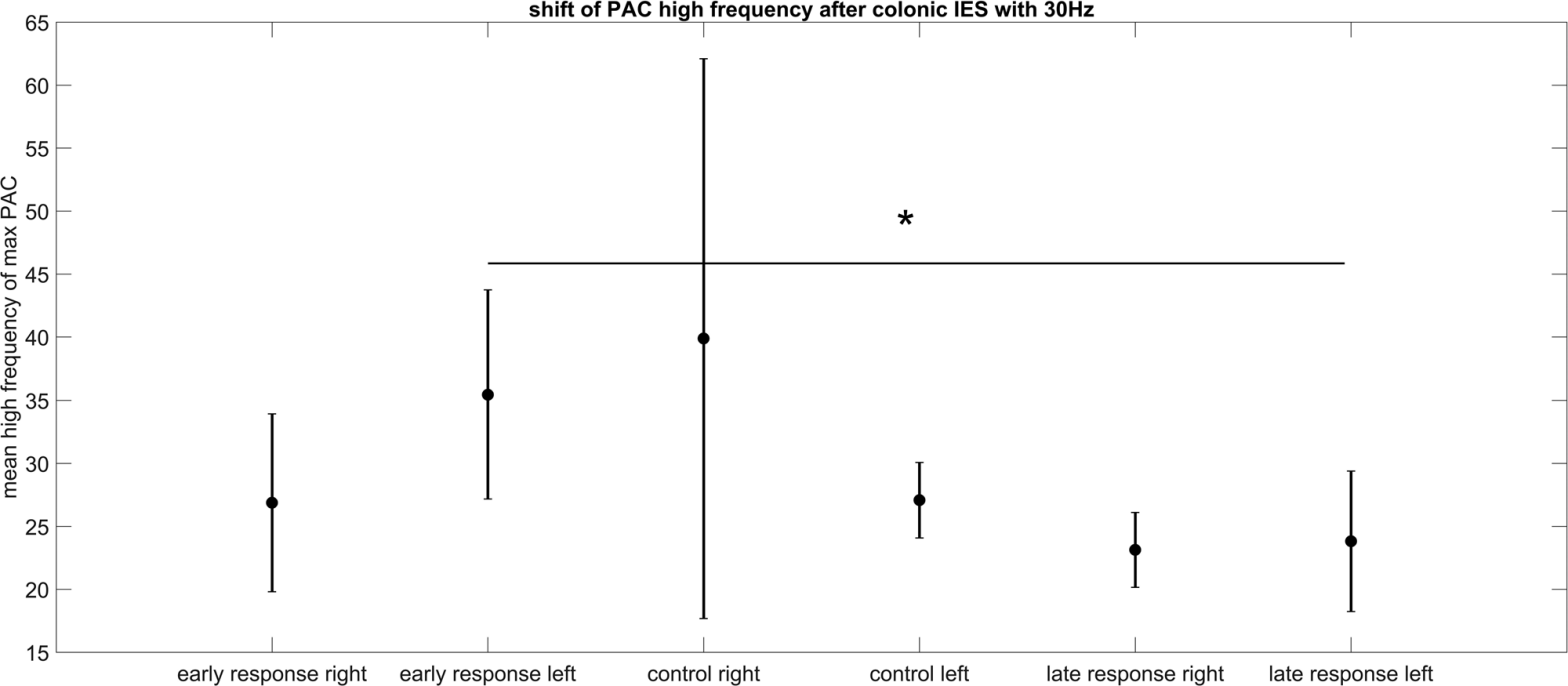
High Frequency of PAC after colon electrical stimulation with 30 Hz. The error bar represents standard deviation. Only for 30 Hz colon stimulation, a statistically significant change in the coupling frequencies of the PAC analysis can be detected. The left hemispheric late response has lower high frequencies for max PAC in comparison to the corresponding early response.

We did not find differences in phase locking value, amplitude envelope correlation, or correlation between the two parietal electrode positions of the EEG after IES. Furthermore, we found only one case (after colon stimulation with 30 Hz, left hemisphere) where the frequency component of the PAC changed between early response and late response (Table 10). In this case, the high frequency component shifted from gamma band in the early response period after IES to the beta band during the late response period after IES (CI 1.41–12.99 and *p* = 0.03) (Figure 5).

**Table 10:** High frequency of max PAC for left parietal electrode position

Group	Group	Lower CI	Estimate	Upper CI	*p*-value
1	2	−1.59	4.20	9.99	0.30
1	3	1.41	7.20	12.99	0.03
2	3	−2.79	3	8.79	0.54

1 = first time window/fast response, 2 = without stimulation, 3 = late response, IES colon (30 Hz frequency).

**Figure 6:**
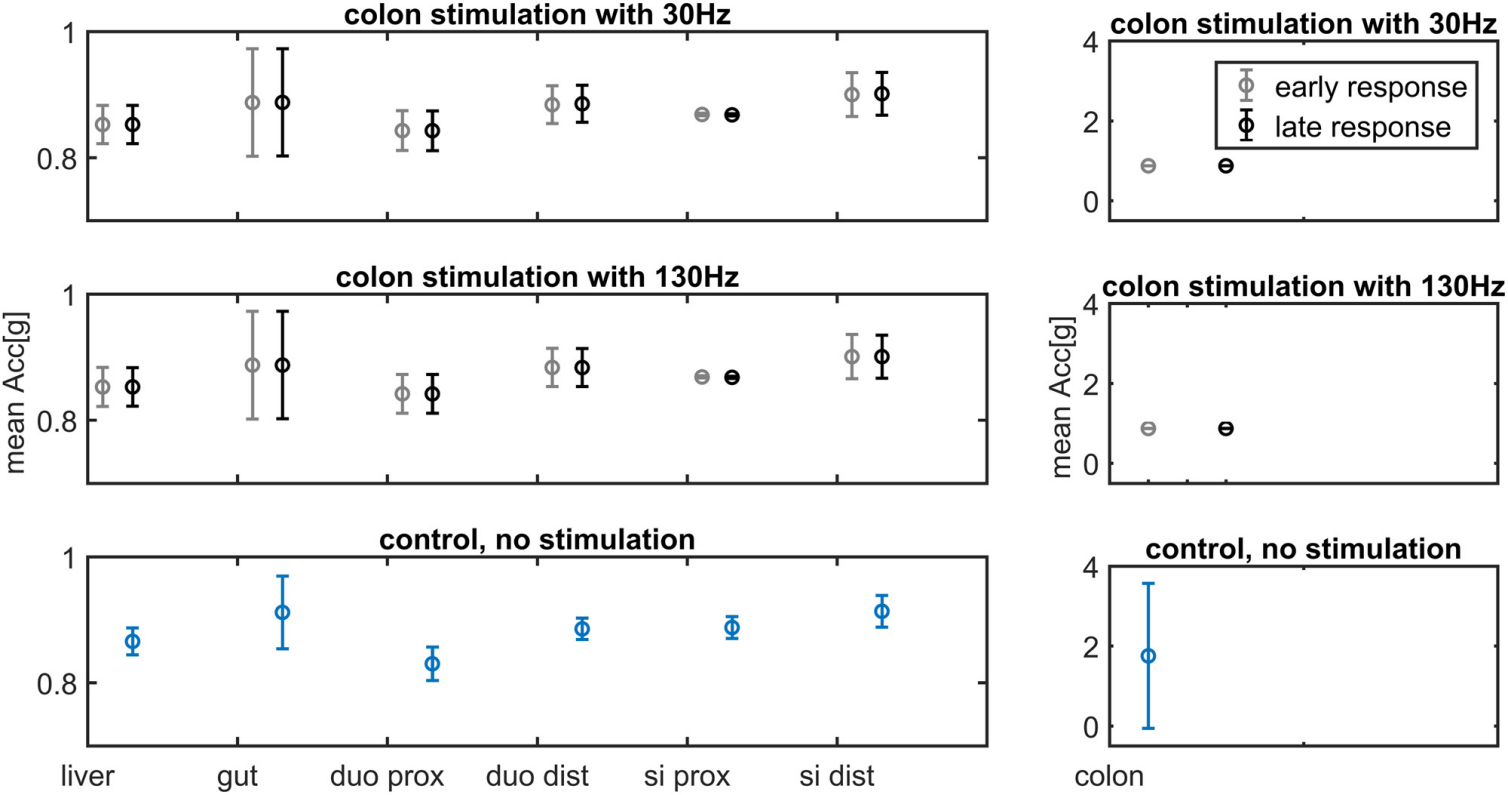
Acceleration measurements at seven different intestinal locations during EEG measurements. In comparison to the control, the early and late response colonic accelerations are decreased. The liver was recorded to detect the impact of artificial ventilation.

**Figure 7:**
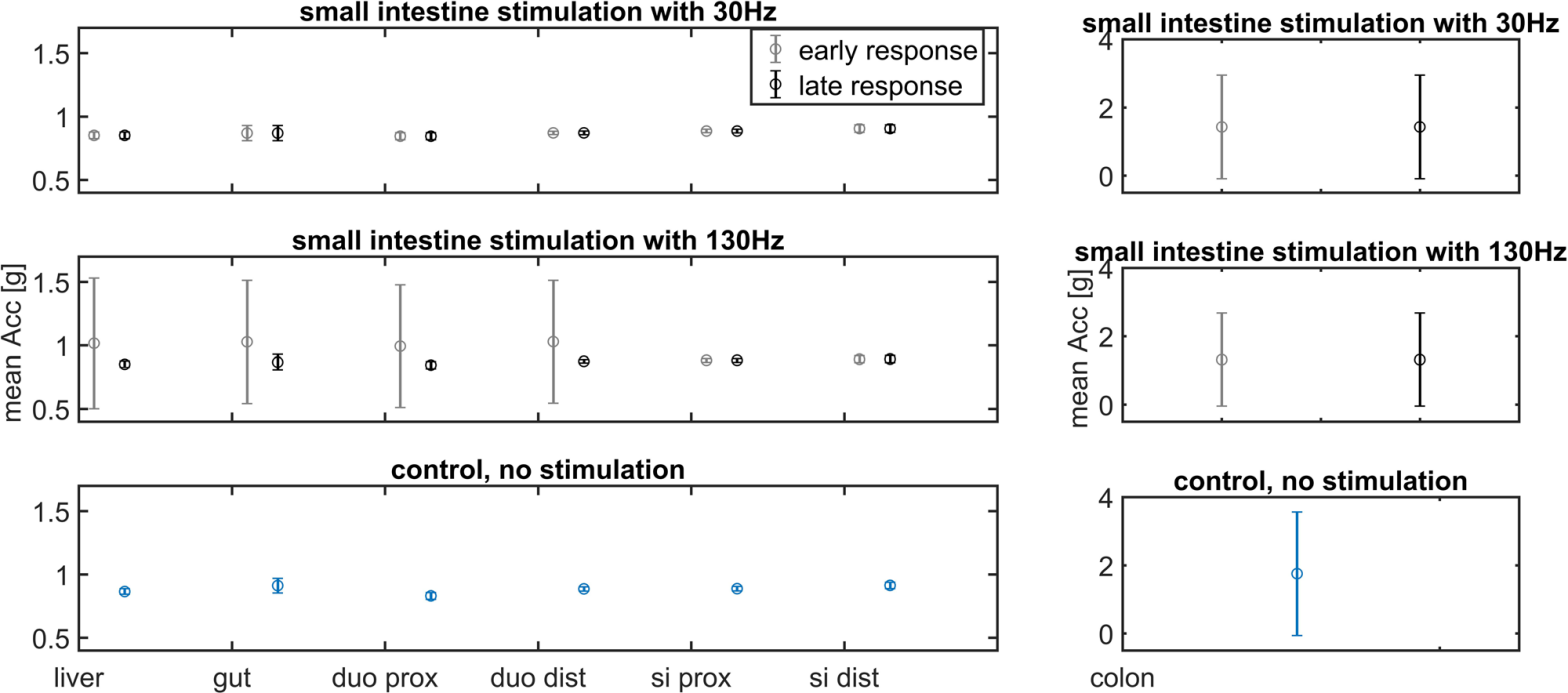
Acceleration after electrical stimulation of the small intestine. Due to the wide range of standard deviation for colonic acceleration, this part is shown in a separate axis (right figure column). The acceleration was continuously measured for seven locations along the gastrointestinal tract. Movements around gut and liver can be influenced by artificial ventilation. Small intestine (Si) stimulation with 130 Hz results in higher acceleration in oral direction (duodenum).

**Figure 8:**
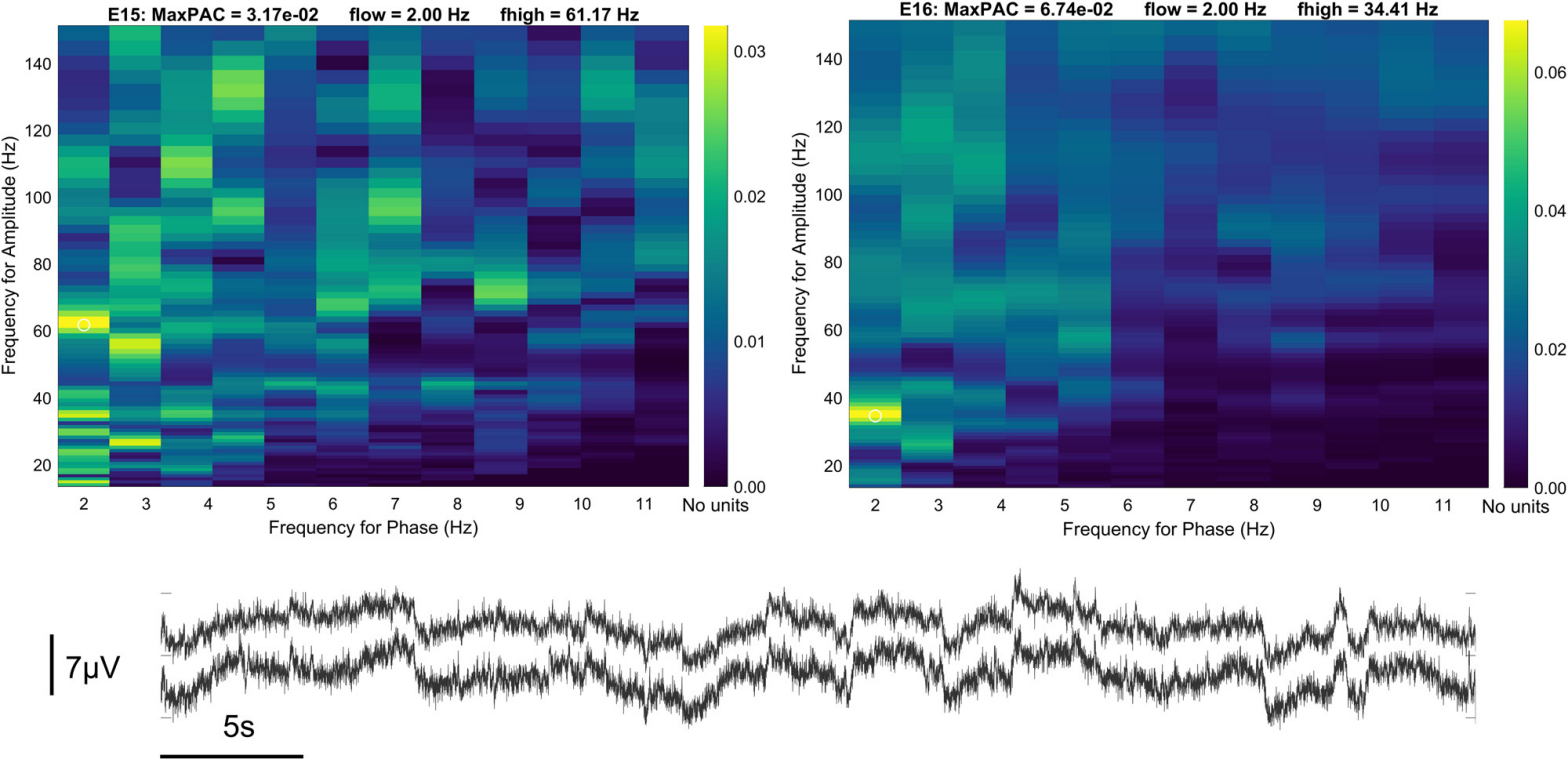
EEG raw data and corresponding PAC maps. E15 (upper left panel) represents PAC map for the right hemispheric EEG recording and E16 (upper right) for the left hemisphere. The corresponding DC EEG raw data trace is shown at the bottom.

The acceleration was continuously monitored throughout the experiments at seven different intestinal locations. After colonic stimulation, the acceleration decreased at the site of stimulation during the early and late response time window of the max PAC analysis (Figure 6). For small intestine stimulation, an increase in acceleration is visible only for the early response and after 130 Hz stimulation in oral direction from the stimulation site (Figure 7). A correlation between PAC and acceleration could not be found. A raw EEG trace and the corresponding PAC maps are shown in Figure 8.

## 4 Discussion

Not only for basic research but also regarding certain diseases, there is growing evidence that the borders between different organs or body compartments are much more fluent than one would assume on the basis of available literature. This knowledge gap is shrinking during recent years, especially because it turned out that some diseases are much more complex and physiologically as well as anatomically widespread than estimated initially. The search term “brain gut axis” reveals 3,143 hits in pubMed.ncbi search engine. Researchers and clinicians are forced to leave their niche in order to understand diseases like Parkinson’s, IBS, and many more. Parkinson’s disease is essentially regarded as CNS disorder with loss of dopaminergic neurons in the substantia nigra. To date, it is still not possible to cure Parkinson’s disease, even if patients can live relatively long with the symptoms. Since longer time, it is known that Parkinson’s disease has also a gastrointestinal (GI) symptomatology, often regarded as side effect of the CNS disorder. Meanwhile some authors hypothesize a possible origin of Parkinson’s in the GI, since these symptoms precede the CNS symptoms by years [6]. Especially, the spread of alpha-synuclein clusters, which are associated with neural loss, from the gut to the brain has been proven in the mouse model recently [34]. On the other hand, IBS is mostly regarded as GI disorder. Here it is the opposite, growing evidence suggests a strong central influence especially with respect to visceral pain [35].

Starting from this practical point of view we designed the underlying question of this study. Nevertheless, it is not trivial to combine central and peripheral physiological measurements. During the past, several authors identified evoked potentials in cortical areas after IES [19,36]. To measure these tiny effects, many repetitions are necessary. This is possible for a scientific setting but much less realistic in the clinical context.

Therefore, we used in this study more robust tools for EEG analysis. PAC is a network-driven phenomenon represented as synchrony dependent amplitude modulation. The amplitude of a faster EEG wave is modulated if it occurs in a certain phase relative to a slower EEG wave. Since these relations are relatively complex and based upon intrinsic network phenomena, they are less prone to artifacts like line noise or movement. We used this kind of analysis for very different questions, ranging from CNS development [37] to the assessment of behavioral states of animals in stress situations [38].

Here we were able to show that a relatively low number of repetitions is necessary to find EEG responses after IES with PAC analysis tools. The response latency is dependent on the nature of the stimulus, which is also true for effects of IES on peristalsis which range from inhibition to the entrainment of peristaltic waves (literature review in the supplementary information). In a former study, we were able to reconstruct the peristaltic movement of our stimulation protocol by means of special accelerometers. It turned out that it was not possible to entrain a peristaltic wave, but instead an inhibitory effect was seen, especially in regions distal (abdominal) to the site of stimulation.

Our results indicate that the IES response regarding peristalsis as well as EEG PAC is specific to certain visceral areas. Generally, in our data PAC is always reduced after IES. After colon stimulation with 30 and 130 Hz, the resulting pattern in the brain is a clear reduction in PAC for the early as well as late response (Figures 3 and 4). The same relatively strong inhibition for the early and late responses as well as 30 and 130 Hz can be seen in case of peristalsis, measured as colon acceleration (Figure 6). Here it can be assumed that the colon region is very sensitive to relatively high stimulus intensities (which are used in this study) which may cover frequency specific stimulation effects, which can therefore not be excluded for a more sophisticated stimulation protocol, as proposed in Table 10. In contrast, for the stimulation of the small intestine, clearly deviant effects can be seen for 30 and 130 Hz stimulation. In case of the 30 Hz stimulation, a very strong reduction in the PAC can be observed especially for the early response (Figure 1), whereas nearly no effect can be seen regarding visceral movement in comparison to a control without stimulation (Figure 7). After stimulation with 130 Hz, an overall reduction in PAC can be observed for the early as well as late response, but additionally a very pronounced increase in variability can be observed, especially for the early response time period, directly after stimulation (Figure 2). Interestingly, a high variability can also be seen for the acceleration of specifically the early response for all visceral measurement points in oral direction to the stimulation site (Figure 7). This effect may indicate a frequency-dependent facilitation of propagation direction. Regarding the frequency components of the PAC, we found nearly no changes for the high and low band after IES with one exception. After colon stimulation with 30 Hz, the high frequency component was slightly elevated, especially for the early response and the left brain hemisphere (Figure 4). We would expect stronger effects in a chronic model system without anesthesia.

To decipher exact relations between brain network responses and IES, a systematic stimulation protocol would be necessary (Table 10 and literature review in the supplementary information), preferentially in a chronic model without anesthesia. Anesthesia has a well-known influence on peristalsis [39] and the EEG pattern [40].

Limitations of this study are the low number of animals (*n* = 3), the acute model system with deep anesthesia and opened visceral cavity as well as dorsal body position during the measurements, a lack of standardized feeding of the animals during the pre-experimental period, and the animal surgery room was not optimized for electrophysiological measurements. Furthermore, a suboptimal stimulation protocol with relatively high stimulation amplitude was used, which has an inhibitory effect on intestinal contractions [1]. 30 mA amplitude have been used in this study. Instead, a stimulation amplitude below 10 mA is common (for example [41]).

A strength of the study is the technically challenging experimental setup with combined measurements of full-band DC EEG and peristalsis during IES as well as EEG analysis with respect to network-driven phenomena.

We propose a much more complex stimulation protocol (Table 11), preferably with a chronic pig model and a higher number of non-invasive EEG electrodes for future experimental settings regarding the interplay between IES and brain activity.

**Table 11:** Proposal of stimulation protocol based on literature review

Frequency (Hz)	Duration	Pulse number	Pulse width	Amplitude
0.2	1 min	12	5 s	100 µA
0.2	10 min	120	5 s	100 µA
0.2	1 min	12	5 s	2 mA
0.2	10 min	120	5 s	2 mA
0.2	1 min	12	5 s	30 mA
0.2	10 min	120	5 s	30 mA
1	10 s	10	1 s	100 µA
1	2 min	120	1 s	100 µA
1	15 min	900	1 s	100 µA
1	10 s	10	1 s	2 mA
1	2 min	120	1 s	2 mA
1	15 min	900	1 s	2 mA
1	10 s	10	1 s	30 mA
1	2 min	120	1 s	30 mA
1	15 min	900	1 s	30 mA
20	1 s	20	50 ms	100 µA
20	1 min	1,200	50 ms	100 µA
20	10 min	12,000	50 ms	100 µA
20	1 s	20	50 ms	2 mA
20	1 min	1,200	50 ms	2 mA
20	10 min	12,000	50 ms	2 mA
20	1 s	20	50 ms	30 mA
20	1 min	1,200	50 ms	30 mA
20	10 min	12,000	50 ms	30 mA
50	1 s	50	20 ms	100 µA
50	1 min	3,000	20 ms	100 µA
50	10 min	30,000	20 ms	100 µA
50	1 s	50	20 ms	2 mA
50	1 min	3,000	20 ms	2 mA
50	10 min	30,000	20 ms	2 mA
50	1 s	50	20 ms	30 mA
50	1 min	3,000	20 ms	30 mA
50	10 min	30,000	20 ms	30 mA
80	1 s	80	12.5 ms	100 µA
80	1 min	4,800	12.5 ms	100 µA
80	10 min	48,000	12.5 ms	100 µA
80	1 s	80	12.5 ms	2 mA
80	1 min	4,800	12.5 ms	2 mA
80	10 min	48,000	12.5 ms	2 mA
80	1 s	80	12.5 ms	30 mA
80	1 min	4,800	12.5 ms	30 mA
80	10 min	48,000	12.5 ms	30 mA
120	1 s	120	8.3333 ms	100 µA
120	1 min	7,200	8.3333 ms	100 µA
120	10 min	72,000	8.3333 ms	100 µA
120	1 s	120	8.3333 ms	2 mA
120	1 min	7,200	8.3333 ms	2 mA
120	10 min	72,000	8.3333 ms	2 mA
120	1 s	120	8.3333 ms	30 mA
120	1 min	7,200	8.3333 ms	30 mA
120	10 min	72,000	8.3333 ms	30 mA

In conclusion of these preliminary study results, EEG PAC analysis may be a fast, non-invasive, and robust tool to study the effect of IES and GI disorders on the level of the CNS. PAC appears to map IES even when no distinct peristalsis is elicited, possibly at a purely somatosensory level. Nevertheless, PAC is not restricted to the CNS but also occurring in the ENS to generate the peristaltic movement. Hence, PAC analysis may also be interesting for a bidirectional analysis of the electrical brain-gut-axis and gut-brain-axis. It may be possible that non-invasive brain stimulation can impact peristalsis and the other way around, that IES can be used as stimulation therapy for certain CNS diseases in the future. Long distance stimulation might resemble natural electrical activity in a better way, because whole networks can be triggered in contrast to local stimulation at the affected disease locations.

## References

[1] Yin J, Chen JDZ. Mechanisms and potential applications of intestinal electrical stimulation. Digestive Dis Sci. 2010;55(5):1208–20. 10.1007/s10620-009-0884-3 19629689

[2] Malek N. Deep brain stimulation in Parkinson’s disease. Neurol India. 2019;67(4):968–78. 10.4103/0028-3886.266268. 31512617

[3] Drobisz D, Damborská A. Deep brain stimulation targets for treating depression. Behav Brain Res. 2018;359:266–73. 10.1016/j.bbr.2018.11.004. 30414974

[4] Carlessi AS, Borba LA, Zugno AI, Quevedo J, Réus GZ. Gut microbiota-brain axis in depression: The role of neuroinflammation. Eur J Neurosci. 2019;00:1–14. 10.1111/ejn.14631. 31785168

[5] Rao M, Gershon MD. The bowel and beyond: the enteric nervous system in neurological disorders. Nat Rev Gastroenterol Hepatol. 2016;13(9):517–28. 10.1038/nrgastro.2016.107. PMC5005185 27435372

[6] Santos SF, de Oliveira HL, Yamada ES, Neves BC, Pereira, Jr A. The Gut and Parkinson’s Disease - A Bidirectional Pathway. Front Neurol. 2019;10:574. 10.3389/fneur.2019.00574. PMC6558190 31214110

[7] Jones MP, Dilley JB, Drossman D, Crowell MD. Brain-gut connections in functional GI disorders: anatomic and physiologic relationships. Neurogastroenterol Motil. 2006;18:91–103. 10.1111/j.1365-2982.2005.00730.x. 16420287

[8] Wehrwein EA, Orer HS, Barman SM. Overview of the anatomy, physiology, and pharmacology of the autonomic nervous system. Compr Physiol. 2016;6:1239–78. 10.1002/cphy.c150037. 27347892

[9] Thomsen L, Robinson TL, Lee JC, Farraway LA, Hughes MJ, Andrews DW, et al. Interstitial cells of Cajal generate a rhythmic pacemaker current. Nat Med. 1998;4(7):848–51. 10.1038/nm0798-848 9662380

[10] Drake MJ, Fowler CJ, Griffiths D, Mayer E, Paton JF, Birder L. Neural control of the lower urinary and gastrointestinal tracts: supraspinal CNS mechanisms. Neurourol Urodyn. 2010;29(1):119–27. 10.1002/nau.20841. 20025025

[11] Konturek SJ, Konturek JW, Pawlik T, Brzozowski T. Brain-gut axis and its role in the control of food intake. J Physiol Pharmacol. 2004;55(1):137–54. 15082874

[12] Huizinga JD, Parsons SP, Chen J-H, Pawelka A, Pistilli M, Li C, et al. Motor patterns of the small intestine explained by phase-amplitude coupling of two pacemaker activities: the critical importance of propagation velocity. Am J Physiol–Cell Physiology. 2015;309(6):C403–14. 10.1152/ajpcell.00414.2014. PMC4572367 26135802

[13] Tort AB, Komorowski R, Eichenbaum H, Kopell N. Measuring phase-amplitude coupling between neuronal oscillations of different frequencies. J Neurophysiol. 2010;104(2):1195–1210. 10.1152/jn.00106.2010. PMC2941206 20463205

[14] Li C, Jacobs D, Hilton T, Del Campo M, Chinvarun Y, Carlen PL, et al. Epileptogenic source imaging using cross-frequency coupled signals from scalp EEG. IEEE Trans Biomed Eng. 2016;63(12);2607–18. 10.1109/TBME.2016.2613936. 27875126

[15] Scheffzük C, Kukushka VI, Vyssotski AL, Draguhn A, Tort ABL, Brankačk J, et al. Selective coupling between theta phase and neocortical fast gamma oscillations during REM-sleep in mice. PLOS ONE. 2011;6(12):28489. 10.1371/journal.pone.0028489. PMC3230633 22163023

[16] Zhang R, Ren Y, Liu C, Xu N, Li X, Cong F, et al. Temporal-spatial characteristics of phase-amplitude coupling in electrocorticogram for human temporal lobe epilepsy. Clin Neurophysiol. 2017;128(9):1707–18. 10.1016/j.clinph.2017.05.020. 28755546

[17] Frieling T, Enck P, Wienbeck M. Cerebral responses eviked by electrical stimulation of rectosigmoid in normal subjects. Digestive Dis Sci. 1989;34(2):202–5. 10.1007/BF01536051 2914538

[18] Loening-Baucke V, Read NW, Yamada T. Cerebral evoked potentials after rectal stimulation. Electroencephalogr Clin Neurophysiol. 1991;80(6):490–5. 10.1016/0168-5597(91)90130-p. 1720724

[19] Garvin B, Lovely L, Tsodikov A, Minecan D, Hong S, Wiley JW. Cortical and spinal evoked potential response to electrical stimulation in human rectum. Worl J Gastroenterol. 2010;16(43):5440–6. 10.3748/wjg.v16.i43.5440. PMC2988236 21086561

[20] Drewes AM, Rössel P, Le Pera D, Arendt-Nielsen L, Valeriani M. Dipolar source modelling of brain potentials evoked by painful electrical stimulation of the human sigmoid colon. Neurosci Lett. 2004;358(1):45–8. 10.1016/j.neulet.2003.12.101. 15016431

[21] Brock C, Olesen SS, Valeriani M, Arendt-Nielsen L, Drewes AM. Brain activity in rectosigmoid pain: unravelling conditioning pain modulatory pathways. Clin Neurophysiol. 2012;123(4):829–37. 10.1016/j.clinph.2011.07.047. 21925938

[22] Lelic D, Olesen AE, Gregersen H, Dahan A, Kolesnikov Y, Drewes AM. Morphine modifies the cingulate-operculum network underlying painful rectal evoked potentials. Neuropharmacology. 2014;77:422–7. 10.1016/j.neuropharm.2013.10.020 Epub 2013 Nov 1. 24184388

[23] Pigarev IN, Bagaev VA, Levichkina EV, Fedorov GO, Busigina II. Cortical visual areas process intestinal information during slow-wave sleep. Neurogastroenterol Motil. 2013;25(3):268–75e169. 10.1111/nmo.12052. 23216826

[24] Kukorelli T, Juhász G. Electroencephalographic synchronization induced by stimulation of small intestine and splanchnic nerve in cats. Electroencephalogr Clin Neurophysiol. 1976;41(5):491–500. 10.1016/0013-4694(76)90061-4. 61853

[25] Rössel P, Pedersen P, Niddam D, Arendt-Nielsen L, Chen ACN, Drewes AM. Cerebral response to electric stimulation of the colon and abdominal skin in healthy subjects and patients with irritable bowel syndrome. Scand J Gastroenterology. 2001;36(12):1259–66. 10.1080/003655201317097092. 11761014

[26] Burgell RE, Lelic DI, Carrington EV, Lunniss PJ, Olesen SS, Surguy SU, et al. Assessment of rectal afferent neuronal function and brain activity in patients with constipation and rectal hyposensitivity. Neurogastroenterol Motil. 2013;25(3):260–7. 10.1111/nmo.12047. Canolty RT, Edwards E, Dalal SS, Soltani M, Nagarajan SS, Kirsch HE, et al. High gamma power is phase-locked to theta oscillations in human neocortex. Science. 2006;313:1626–28. 10.1111/nmo.12047. 23240734

[27] Graversen C, Brock C, Drewes AM, Farina D. Biomarkers for visceral hypersensitivity identified by classification of electroencephalographic frequency alterations. J Neural Eng. 2011;8(5):056014. 10.1088/1741-2560/8/5/056014. Epub 2011 Sep 15. 21918294

[28] Dimcevski G, Sami SA, Funch-Jensen P, Le Pera D, Valeriani M, Arendt-Nielsen L, et al. Pain in chronic pancreatitis: the role of reorganization in the central nervous system. Gastroenterology. 2007;132(4):1546–56. 10.1053/j.gastro.2007.01.037. 17408654

[29] Lo Y-K, Wang PM, Dubrovsky G, Wu MD, Chan M, Dunn J, et al. A wireless implant for gastrointestinal motility disorders. Micromachines. 2017;9(17):2–13. 10.3390/mi9010017. PMC6187657 30393295

[30] de Camp NV, Heimann A, Kempski O, Bergeler J. Accelerometer-based assessment of intestinal peristalsis: Toward miniaturized low-power solutions for intestinal implants. IEEE J Transl Eng Health Med. 2018;6:2700507. 10.1109/JTEHM.2018.2864975. PMC6147734 30245946

[31] Sauleau P, Lapouble E, Val-Laillet D, Malbert C-H. The pig model in brain imaging and neurosurgery. Ainaml. 2009;3(8):1138–51. 10.1017/S1751731109004649. 22444844

[32] Tadel F, Baillet S, Mosher JC, Pantazis D, Leahy RM. Brainstorm: a user-friendly application for MEG/EEG analysis. Comput Intell Neurosci. 2011;879716(3):1–13. 10.1155/2011/879716. PMC3090754 21584256

[33] Canolty RT, Knight RT. The functional role of cross-frequency coupling. Trends Cogn Sci. 2010;14(11):506–15. 10.1016/j.tics.2010.09.001. PMC3359652 20932795

[34] Kim S, Kwon SH, Kam TI, Panicker N, Karuppagounder SS, Lee S, et al. Transneuronal Propagation of Pathologic α-Synuclein from the Gut to the Brain Models Parkinson’s disease. Neuron. 2019;103(4):627–41.e7. 10.1016/j.neuron.2019.05.035. PMC6706297 31255487

[35] Moloney RD, Johnson AC, O'Mahony SM, Dinan TG, Greenwood-Van Meerveld B, Cryan JF. Stress and the Microbiota–Gut–Brain Axis in Visceral Pain: Relevance to Irritable Bowel Syndrome. CNS Neurosci Ther. 2016;22(2):102–17. 10.1111/cns.12490. PMC6492884 26662472

[36] Rössel P, Arendt-Nielsen L, Niddam D, Chen AC, Drewes AM. Short latency cerebral response evoked by painful electrical stimulation applied to the human sigmoid colon and to the convergent referred somatic pain area. Exp Brain Res. 2003;151:115–22. 10.1007/s00221-003-1484-7. 12712308

[37] de Camp NV, Hense F, Lecher B, Scheu H, Bergeler J. Models for preterm cortical development using non invasive clinical EEG. Transl Neurosci. 2017;8:211–24. 10.1515/tnsci-2017-0029. PMC5811640 29445543

[38] de Camp NV, Ladwig-Wiegard M, Geitner CI, Bergeler J, Thöne-Reineke C. EEG based assessment of stress in horses: a pilot study. PeerJ. 2020;8:e8629. 10.7717/peerj.8629. eCollection 2020. PMC7227666 32435527

[39] Desmet M, Vander Cruyssen P, Pottel H, Carlier S, Devriendt D, Van Rooy F, et al. The influence of propofol and sevoflurane on intestinal motility during laparoscopic surgery. Acta Anaesthesiol Scand. 2016;60(3):335–42. 10.1111/aas.12675 26806956

[40] Constant I, Sabourdin N. The EEG signal: a window on the cortical brain activity. Pediatr Anesthesia. 2012;22:539–52. 10.1111/j.1460-9592.2012.03883.x. 22594406

[41] Dong Y, Yin J, Zhang Y, Chen JDZ. Electronic bypass for diabetes: Optimization of stimulation parameters and mechanisms of glucagon like peptide-1. Neuromodulation. 2021. 10.1111/ner.13367. 33538043

